# When the Trojan horse is unable to reach inside the city: investigation of the mechanism of resistance behind the first reported cefiderocol-resistant *E. coli* in Canada

**DOI:** 10.1128/spectrum.03223-23

**Published:** 2024-03-25

**Authors:** Kevin R. Barker, Gabriel W. Rebick, Ken Fakharuddin, Clayton MacDonald, Michael R. Mulvey, Laura F. Mataseje

**Affiliations:** 1 Microbiology, Department of Laboratory Medicine and Genetics, Trillium Health Partners, Mississauga, Ontario, Canada; 2 Department of Laboratory Medicine and Pathobiology, University of Toronto, Toronto, Ontario, Canada; 3 Institute for Better Health, Trillium Health Partners, Mississauga, Ontario, Canada; 4 Division of Infectious Diseases, Department of Medicine, Trillium Health Partners, Mississauga, Ontario, Canada; 5 Public Health Agency of Canada, National Microbiology Laboratory, Winnipeg, Canada; University of Pittsburgh School of Medicine, Pittsburgh, Pennsylvania, USA

**Keywords:** antimicrobial resistance, Canada, carbapenemase, carbapenem-resistant *Enterobacteriaceae*, cefiderocol, extensively drug-resistant, ST167, XDR

## Abstract

Gram-negative metallo-β-lactamase-producing bacteria can be extremely problematic, especially when found to be extensively drug-resistant (XDR). Cefiderocol is a novel antimicrobial that has been shown to overcome most carbapenemases, with very rare resistance reported to date. Within our institution, two multidrug-resistant and one XDR strains were isolated from a patient who recently emigrated from India. Each isolate underwent whole-genome sequencing to resolve plasmids and determine phylogenetics, strain typing, and mechanisms of resistance. The XDR *E. coli* was ST167, harbored NDM-5, *cirA* and PBP3 mutations, consistent with cefiderocol resistance. Our study suggests that the NDM region is required in conjunction with *cirA* and PBP3 mutations. It is not clear why; however, our study did determine a potential novel iron-transport region unique to the cefiderocol-resistant isolate. This is the first characterized cefiderocol-resistant *E.coli* reported from Canada. Health centers should be on alert for this clone.

**IMPORTANCE:**

The development of cefiderocol, a novel siderophore cephalosporin, has provided additional options to the treatment of extensively drug-resistant (XDR) Gram-negative bacteria. Resistance to cefiderocol is poorly understood and only recently described. Here, we describe a case of a patient with recent travel to India harboring three *Escherichia coli* isolates, one resistant and two susceptible to cefiderocol. Two isolates are highly similar genetically, allowing the mechanism of resistance to be described more closely. The importance of this manuscript contributes both globally to the understanding of cefiderocol resistance in *E. coli* as well as nationally as this is the first resistant case reported in Canada. This is especially concerning as cefiderocol is not currently approved in Canada. The implications of reporting emerging resistance to new antimicrobials for XDR Gram negatives are impactful to infectious disease specialists, clinical microbiologists, physicians, and public health.

## OBSERVATION

A 77-year-old man with a past medical history of chronic obstructive pulmonary disease, diabetes mellitus, and jaw cancer recently immigrated to Canada from the Punjab province of India, where he had been hospitalized for 6 months. During his hospitalization in India, he received a number of oral antimicrobials in the time leading up to emigration for various complications due to cancer treatment. We are unable to verify, but do not believe that he received any prior cefiderocol. In Canada, he received ceftriaxone and azithromycin for community-acquired pneumonia. One month later, he presented to the hospital with complaints of weakness and confusion, which worsened over 2 days. He had severe hypercalcemia, which was improved with pamidronate and intravenous fluids. *Escherichia coli* was cultured from urine and blood, and the admission screen was positive for an *E. coli* carbapenemase-producing Enterobacterales (CPE), deemed as community-acquired. Fosfomycin was initiated for possible urinary tract infection, with initial clinical improvement. Antimicrobial susceptibility testing using Phoenix (BD, Mississauga, ON, Canada), broth microdilution (Sensititre, Thermo Fisher, Ottawa, ON, Canada) and NG-Test CARBA-5 lateral flow immunoassay (NG-BIOTECH, Guipry, France) results indicated the following: (i) XDR NDM- CPE, only susceptible to fosfomycin (isolate 1); (ii) MDR (carbapenem-susceptible) Enterobacterales (isolate 2); and (iii) OXA-48-like CPE, susceptible to fosfomycin and trimethoprim–sulfamethoxazole (isolate 3), see Table 1 (1). He was switched to a combination treatment of fosfomycin, trimethoprim–sulfamethoxazole, and tigecycline, with clinical response. Further reference laboratory testing of the *E. coli* NDM-CPE by broth microdilution and gradient and disc diffusion revealed resistance to cefiderocol with an MIC of ≥256 mg/L, see Table 1 for results and methods.

**TABLE 1:** Antibiogram of three strains of *E. coli* isolated from the patient’s urine and blood cultures^*d*^

Isolate	Source	Whole-genome sequencing^*a*^					Antimicrobial susceptibility testing (AST) Interpretation^*b*^ (mg/L)																	
		Genotype	Additional resistant genes	Plasmid types	PBP3	cirA																		
							AMC	CT	CZA	IMR	TZP	CRO	ATM	ETP	MEM	GM	TM	AN	CIP	SXT	Fos^*c*^	FDC		
																						DD	GD	BMD
1	Urine	NDM-5	aadA2 bla_CMY-145_ bla_TEM1B_ dfrA12 erm(B) mph(A) qacE rmtB sul1 gyrA (D87N; S83L), parC (S80I; S458A)	IncFIA/FIB/FII (89 Kb) IncI (70 Kb)	YRIN	Stop codon AA 105	R (>16/8)	R (>8/4)	R(>16/4)	R(>8/4)	R(>64/4)	R(>32)	R(>16)	R(>2)	R(>16)	R(>8)	R(>8)	R(>32)	R(>2)	R(>4/76)	S(0.5)	R(n/a)	R(>256)	R (>128)
2	Blood	−	bla_CMY-145_ erm(B) mph(A), gyrA(D87N; S83L) parC (S80I; S458A)	IncFIA/FIB/FII (67 Kb) IncI (70 Kb) IncHI1B/FIB (210 Kb)	YRIN	Stop codon AA 105	R (>16/8)	R(>8/4)	S(≤4/4)	S (≤1/4)	R (>64/4)	R(>32)	R (>16)	I (1)	S (≤0.06)	S (≤2)	S (≤2)	S (≤8)	R (>2)	S (≤2/38)	S (0.5)	S (n/a)	S (4)	S (4)
3	Blood	OXA-181	bla_CMY-42_ bla_TEM1B_ mph(A) qnrS1 rmtB tet(A) gyrA (D87N;S83L) parC (S80I; S458A)	Col-type (4.7 Kb,2Kb) IncFIA/FII(112 Kb) IncI (38 Kb) IncX3/colKP3 (51 Kb)	YRIN	Functional	R (>16/8)	R (>8/4)	S (≤4/4)	S (≤1/4)	R (>64/4)	R (>32)	R (16)	R (>2)	S (0.5)	R (>8)	R (>8)	R (>32)	R (>2)	S (≤2/38)	S (0.5)	S (n/a)	S (0.5)	S (1)

^*a*^ Whole-genome sequencing undertaken using Illumina NextSeq.

^*b*^ AST undertaken by broth microdilution (BMD) unless stated otherwise. FDC AST was done with a commercial BMD kit (ComASP, Liofilchem), gradient diffusion (GD) (Liofilchem) on Mueller–Hinton Agar (MHA), and disc diffusion (DD) (Liofilchem) using 30-µg disks on MHA Interpretation of breakpoints based on the Clinical and Laboratory Standards Institute M100: performance standards for antimicrobial susceptibility testing, 32nd edition.

^*c*^ Susceptibility undertaken using agar dilution.

^*d*^ AMC – amoxicillin-clavulanic acid; AN – amikacin; ATM – aztreonam; CIP – ciprofloxacin; CRO – ceftriaxone; CT – ceftolozane-tazobactam; CZA – ceftazidime-avibactam; ETP – ertapenem; FDC – cefiderocol; FOS – fosfomycin;; GM – gentamicin; I – intermediate; IMR – imipenem-relebactam; MEM – meropenem; n/a – not applicable; R – resistant; S – susceptible; SXT – trimethoprim-sulfamethoxazole; TM – tobramycin; TZP – piperacillin-tazobactam.

Gram-negative bacteria encoding metallo-β-lactamases like NDMs are problematic, as they hydrolyze all known β-lactams except monobactams, including β-lactam-inhibitor combinations like ceftolozane–tazobactam and ceftazidime–avibactam (2–4). Cefiderocol is a siderophore-conjugated cephalosporin, with a novel mechanism of action that can overcome most CPEs by utilizing the intrinsic iron transport systems in bacteria, acting as a “Trojan horse” (5, 6). XDR-Enterobacterales are rare, and here we report the first Canadian case of cefiderocol resistance. There have been a few case reports of cefiderocol-resistant NDM-5-producing *E. coli* worldwide, belonging to the high-risk ST167 clone, which are associated with increased *bla_NDM-5_* copies, a penicillin-binding protein 3 (PBP3) insertion mutation, and an iron-catecholate outer membrane transporter (*cirA*) mutation, through a pre-mature stop codon at amino acid 105 (7–9).

All three isolates underwent whole-genome sequencing using the Illumina and Nanopore platforms to resolve plasmids and determine the phylogenetics (https://github.com/phac-nml/snvphyl-galaxy) and strain typing and mechanisms of resistance (https://github.com/phac-nml/staramr); details in supplemental materials. Both isolate 1 and 2 belonged to ST167; however, only isolate one harbored *bla*_NDM-5_. When comparing phylogenetic relatedness, we observed 8-SNV differences in the core genome, which contained 99% of the total chromosomal content. ST167 has been described as a high-risk clone, associated with *bla*_NDM-5_ (10) and more recently reported in cefiderocol-resistant isolates (7, 8, 11). Isolate 3 belonged to an unrelated ST2659 and harbored *bla*_OXA-181_. We examined the mechanism of cefiderocol resistance by investigating the targets PBP3 (*ftsl*; the primary target of cefiderocol) and *cirA,* previously reported with this phenotype (8, 11). Interestingly, all three isolates harbored a 4-amino acid (YRIN) insertion mutation at amino acid 334 of PBP3. Both ST167 isolates harbored a 2-bp deletion at amino acid 89 in *cirA*, which led to a frameshift mutation and introduction of a pre-mature stop codon at amino acid 105. Isolate 3 (ST2659) harbored a functional *cirA*.

The association of PBP3 and *cirA* with cefiderocol resistance has been described previously. However, in our isolates, the YRIN insertion mutation in PBP3 and *cirA* deletion were not unique to the cefiderocol-resistant isolate (isolate 1). Recent studies have shown that PBP3 mutation may not serve as the primary cause for resistance (8, 9, 11). This agrees with our data, whereby isolate 3 (PBP3 mutation only) had a cefiderocol MIC of 0.5 mg/L (susceptible) and isolate 2 (PBP3 and *cirA* mutations) had an MIC of 4 mg/L (susceptible), suggesting that *cirA* mutations, even with the PBP3 mutation, may not be sufficient to lead to cefiderocol resistance. Only isolate 1 (PBP3, *cirA* mutations, and *bla*_NDM-5_) had an MIC of >256 mg/L (resistant).

**Fig 1:**
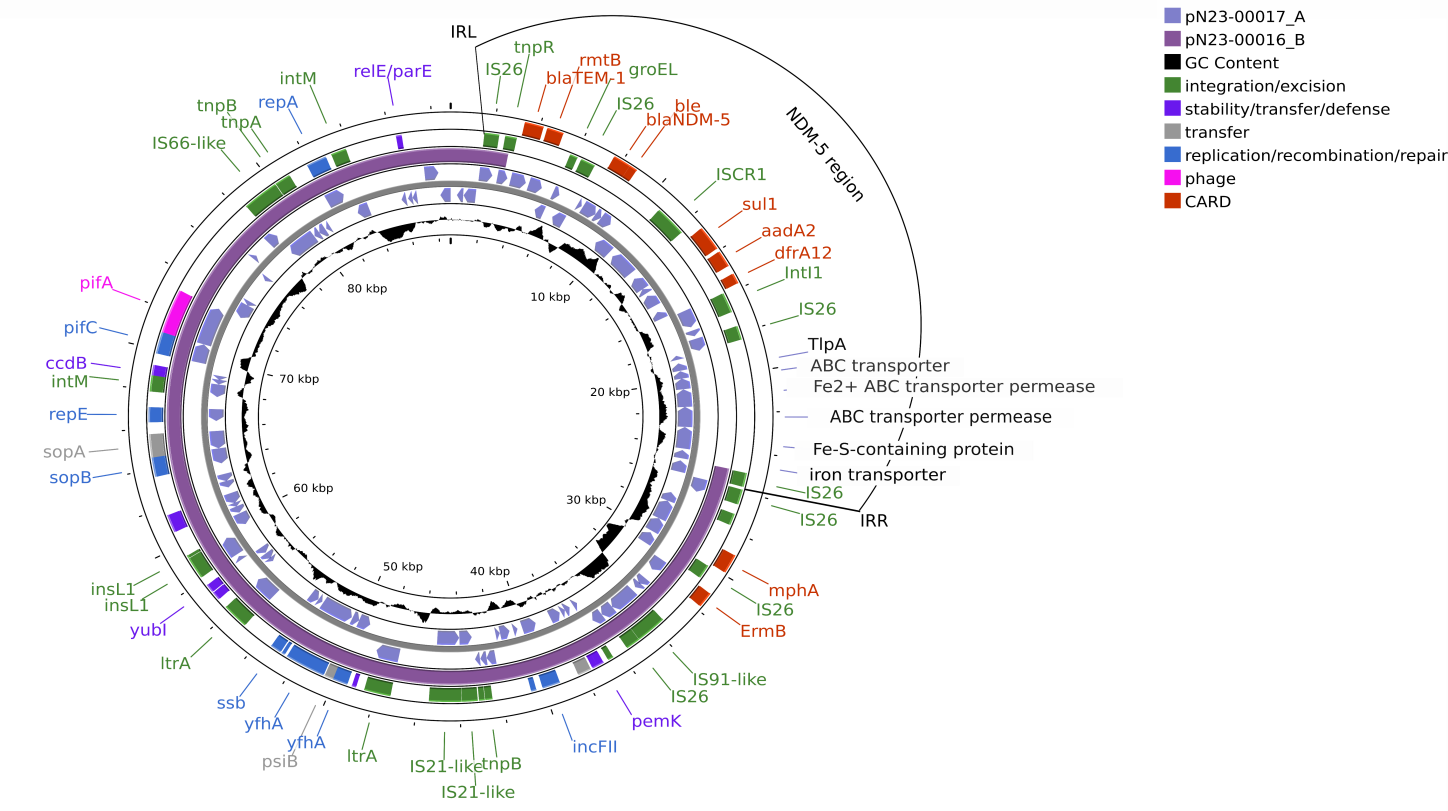
Schematic representation of IncFIA/FIB(AP001918)FII plasmids in isolate 1 (bla_NDM-5_ containing) and isolate 2. The NDM-5 region is characterized by flanking IS26 and 51 bp inverted repeat left (IRL) and right (IRR). Visualization of plasmids was done using Proksee (https://proksee.ca/). Arrows within the inner track represent the direction of CDSs. Proksee identified resistance genes using CARD resistance gene finder and mobile genetic elements using mobileOG-db.

When further comparing the two ST167 isolates, we found an intact 67-Kb phage containing 93 proteins corresponding to PHAGE_Salmon_vB_SosS_Oslo_NC_ 018279 (http://phaster.ca/) present in only the cefiderocol-resistant isolate (isolate 1). Plasmid content was similar with both ST167 isolates, harboring an IncI 70 Kb CMY-145 plasmid. Only isolate 2 harbored a large 210-Kb IncFIB(pNDM-mar)/HI1B(pNDM-mar) plasmid containing no AMR genes. Both isolates contained an IncFIA/FIB(AP001918)/FII plasmid harboring *mph*(A) and *erm*(B); however, only the cefiderocol-resistant isolate (isolate 1) contained the NDM-5 region (Fig. 1). This 24-Kb region is flanked by IS26 and 51 bp inverted repeats, contained AMR genes (*aadA2*, *bla*_NDM-5_, *bla*_TEM1B_, *dfrA12*, *rmtB*, and *sul1*), and interestingly, numerous proteins involved in iron transport. We hypothesize that isolate 2 lost this region through a truncated transposase (Tn3-family) immediately downstream of the IRL and IS26. These findings along with those of other studies suggest that the contribution of PBP3 and *cirA* mutations in conjunction with presence of NDM-5 is associated with cefiderocol resistance; however, it is unclear as to why its presence is necessary to elicit resistance (8, 9).

Our study also found an iron transport region downstream of the NDM region, which may be contributing to cefiderocol resistance. This ~7 kb region contained proteins for both an ABC and iron transporter, both an ABC and iron transporter permease, and an Fe–S-containing protein. We hypothesize that it may (i) act as a competitive iron transport system, facilitating iron acquisition and outcompeting the system associated with cefiderocol binding and transport or (ii) may be an operon that regulates the system being used by the siderophore or cefiderocol-associated transport system. However, further proteomic and expression studies would be required to investigate this.

This is the first reported case and fully characterized isolate of cefiderocol resistance within Canada, and the high-risk clone, ST167. This is also the first report of a possible association of an iron-associated region within the NDM-5 region that may be required for cefiderocol resistance. However, further investigations are required to elucidate the role of this region with cefiderocol resistance. Cefiderocol is not Health Canada-approved and is only available through a special access program. It is therefore likely to be underutilized for the treatment of XDR Enterobacterales, and thus under-tested, exacerbated by the complexity of cefiderocol susceptibility testing. Our data indicate that cefiderocol resistance was associated with mutations in both PBP3 and *cirA* in *E. coli* harboring *bla*_NDM-5_. Further work is required to determine the contribution of the NDM-5 region to resistance. This patient had several risk factors, including prior hospitalization abroad (India), several courses of antimicrobials prior to emigration from India, and prior hospitalization in Canada with antimicrobial treatment. Although cefiderocol resistance remains rare, this may be detrimental to patient care and public health, and so providers must be on high alert.

## Data Availability

All sequence data and assembled genomes are available under BioProject PRJNA971158.

## SUPPLEMENTAL MATERIAL

Supplemental material spectrum.03223-23-s0001.docx Description of the whole genome sequencing methods used.

ASM does not own the copyrights to Supplemental Material that may be linked to, or accessed through, an article. The authors have granted ASM a non-exclusive, world-wide license to publish the Supplemental Material files. Please contact the corresponding author directly for reuse.
