# Supplementary material for: When the Trojan horse is unable to reach inside the city: investigation of the mechanism of resistance behind the first reported cefiderocol-resistant E. coli in Canada
Source: Microbiol Spectr. 2024 Mar 25;12(5):e03223-23. doi: 10.1128/spectrum.03223-23 (PMC11064503; doi:10.1128/spectrum.03223-23)
Supplement: Supplemental material — Description of the whole genome sequencing methods used. [file spectrum.03223-23-s0001.docx]

# Supplemental Material

## Whole genome sequencing methods:

Genomic DNA was extracted using the Epicentre MasterPure Complete kits (Mandel Scientific, Guelph, ON, Canada) following manufacturer's protocols. Short-read sequencing was conducted on the Illumina NextSeq 2000 platform using NexteraXT libraries. Average coverage was >50X. Long-read sequences were generated using the Rapid Barcoding Kit (SQK-RBK004) following manufacturer's protocols on R9.4.1 flow cells with the MinION Mk1B (ONT, Oxford, Oxfordshire, UK). Read data was basecalled and demultiplexed with Guppy v6.4.6 using the Super High Accuracy model (ONT) and default settings. Average ONT depth of coverage was 91X. The assembly workflow was managed using Snakemake [https://doi.org/10.12688/f1000research.29032.2]. ONT reads were trimmed with Porechop v0.2.3_seqan2.1.1 [https://github.com/rrwick/Porechop] and filtered for Q-score > 8 and length > 1000 bases with Filtlong v0.2.1 [https://github.com/rrwick/Filtlong]. Illumina reads had adapters trimmed and were filtered for an average Q-score > 30 with trim-galore v0.6.7 [https://github.com/FelixKrueger/TrimGalore]. FastQC v0.11.9 [http://www.bioinformatics.babraham.ac.uk/projects/fastqc] and Nanoplot v1.28.2 [https://github.com/wdecoster/NanoPlot] were used to assess quality control metrics for Illumina and ONT reads respectively. Isolates were assembled with hybrid Unicycler v0.5.0 using default settings [https://journals.plos.org/ploscompbiol/article?id=10.1371/journal.pcbi.1005595], and were polished with short reads using Polypolish v0.5.0 [https://journals.plos.org/ploscompbiol/article?id=10.1371/journal.pcbi.1009802] and POLCA from MaSuRCA v4.0.9 [https://doi.org/10.1093/bioinformatics/btt476].
